# Insect Freeze-Tolerance Downunder: The Microbial Connection

**DOI:** 10.3390/insects14010089

**Published:** 2023-01-13

**Authors:** Mary Morgan-Richards, Craig J. Marshall, Patrick J. Biggs, Steven A. Trewick

**Affiliations:** 1 Wildlife & Ecology Group, School of Natural Sciences, Massey University Manawatu, Palmerston North 4410, New Zealand; 2 Department of Biochemistry, University of Otago, Dunedin 9016, New Zealand; 3 Molecular Biosciences, School of Natural Sciences, Massey University Manawatu, Palmerston North 4410, New Zealand

**Keywords:** alpine insects, Aotearoa, *Celatoblatta*, freeze tolerance, *Hemideina*, genomics, gut microbes, ice-nucleating agent, microbiome, *Sigaus*

## Abstract

**Simple Summary:**

The alpine insect fauna of Aotearoa/New Zealand is unusual in that many species survive freezing solid at any time of the year. The physiological mechanisms that operate are not well-understood, but we do know the challenges imposed on cells when liquid water expands to form a solid (ice). Insects that are freeze tolerant start freezing at relatively warm temperatures with the help of ice nucleating agents that foster the slow formation of ice crystals outside cells. Here we consider the potential role of microbes living in the guts of alpine Aotearoa/New Zealand insects to facilitate this widespread ability to tolerate freezing. Many bacteria and fungi produce ice-nucleating agents that mediate formation of ice crystals at temperatures just below 0 °C. Some encourage ice formation on foliage with subsequent tissue damage, allowing microbial access to plant nutrients and causing crop damage. Other roles for ice-nucleating agents may include more positive outcomes for insects by facilitating controlled freezing at relatively warm temperatures.

**Abstract:**

Insects that are freeze-tolerant start freezing at high sub-zero temperatures and produce small ice crystals. They do this using ice-nucleating agents that facilitate intercellular ice growth and prevent formation of large crystals where they can damage tissues. In Aotearoa/New Zealand the majority of cold adapted invertebrates studied survive freezing at any time of year, with ice formation beginning in the rich microbiome of the gut. Some freeze-tolerant insects are known to host symbiotic bacteria and/or fungi that produce ice-nucleating agents and we speculate that gut microbes of many New Zealand insects may provide ice-nucleating active compounds that moderate freezing. We consider too the possibility that evolutionary disparate freeze-tolerant insect species share gut microbes that are a source of ice-nucleating agents and so we describe potential transmission pathways of shared gut fauna. Despite more than 30 years of research into the freeze-tolerant mechanisms of Southern Hemisphere insects, the role of exogenous ice-nucleating agents has been neglected. Key traits of three New Zealand freeze-tolerant lineages are considered in light of the supercooling point (temperature of ice crystal formation) of microbial ice-nucleating particles, the initiation site of freezing, and the implications for invertebrate parasites. We outline approaches that could be used to investigate potential sources of ice-nucleating agents in freeze-tolerant insects and the tools employed to study insect microbiomes.

## 1. Introduction

Within their natural ranges, organisms experience temperatures that fluctuate daily and seasonally with varying degrees of predictability [1]. Life cycles are often strongly tied to the changing seasons as resources and conditions shift. Species living at higher latitudes are exposed to greater temperature variation through their lives than those living close to the equator. Those that are ectotherms, including insects, are generally unable to maintain an optimal internal temperature independent of ambient conditions, so when environmental temperatures fall toward the freezing point of water, insect activity reduces and eventually stops. In temperate regions, insect life cycles are tuned to the seasons with overwintering dormancy typically at a specific developmental stage, such as egg or pupa [2]. Winter dormancy involving diapause is the predominant survival strategy among insect species in temperate and boreal regions of the northern hemisphere. Diapause is a genetically programmed developmental hiatus that cannot be immediately terminated by the return of favourable conditions, so its evolution is linked to environmental cyclicity [3]. For example, *Melanoplus sanguinipes* grasshoppers in North America overwinter as eggs that enter diapause depending on population-specific environmental conditions [4]. The range of responses to cold is broad in insects [5] but species can be loosely categorized into those that cannot tolerate any ice in their bodies (freeze-intolerant), and those that survive ice (freeze-tolerant). Cold- or freeze-intolerant insects either die when exposed to low temperatures or have mechanisms to prevent ice crystallisation and its damage (freeze avoidance).

Many insects couple diapause with freeze-avoidance mechanisms in their lifecycle by producing ‘anti-freeze’ proteins to defer mortality as winter approaches. These proteins, such as the β-helical protein of spruce budworm larvae [6], suppress the temperature at which water in and around their cells forms ice crystals. Others have evolved a different strategy that allows freeze-tolerance; the ability to survive freezing. Among pterygote insects that have been studied, most species (>70%) that are able to survive cold temperatures do so by freeze avoidance [7]. Many of the well-studied species are in high-latitude landscapes of the northern hemisphere, where they are exposed to extreme but predominantly seasonal temperature change [8,9]. In Aotearoa/New Zealand, however, where a relatively mild maritime temperate climate appears to result in relaxed diapause [9], the prevalent (85%) strategy among endemic mountain invertebrates is to freeze solid when temperatures dip below 0 °C [10]. In the Southern Alps of New Zealand, sub-zero temperatures can occur at any time of year, so these insects freeze at any time of the year and growth stage then reanimate as their environment warms [10].

**Figure 1:**
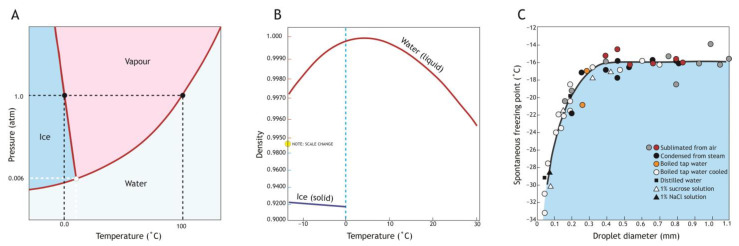
The metric temperature scale introduced by Anders Celsius is based on prominent phase changes of water; freezing at 0 °C and boiling at 100 °C at 1 atmosphere pressure. (**A**) Water phase changes, solid from vapour, vapour from liquid, and liquid from solid, reflect temperature and pressure. These meet at a single point called the triple point, where all three phases can coexist. (**B**) However, supercooling of water occurs when temperature reduction below 0 °C is not accompanied by crystallization. In biological systems the formation of ice decreases the density by nearly ~9%, leading to an increase in volume of the crystalline structure (ice) that can disrupt tissues. Density changes are illustrated over the ~40 °C range likely to be experienced by New Zealand alpine insects. (**C**) Droplet size influences the temperature at which water phase change occurs (liquid to ice) as demonstrated by Heverly (1949) [14] who cooled water droplets of different sizes to measure their temperature of crystallization, and noted that at this scale the purity of the water used had little effect (colours indicates origin of water).

Water is an amazing molecule that forms the matrix of life on Earth [11] having unusual and biologically significant molecular properties [12]. As a liquid, it contains a multitude of hydrogen bonds between O and H atoms of adjacent molecules that are in rapid flux. The density of liquid H_2_O varies with temperature; it is maximal as a liquid at about 4 °C and declines when either heated or cooled. Critically, water undergoes negative thermal expansion when it freezes because the open and ordered hexagonal crystalline structure of ice is less dense (occupying ~9% more volume) than liquid water, and so floats (Figure 1) [13].

In ambient conditions, the highest temperature at which water can freeze is 0 °C, but at this temperature pure water is unlikely to undergo the phase change to a crystalline form that we usually consider to be ‘freezing’. The minimum temperature at which ice can form is suppressed by the presence of certain solutes (resulting in freezing point depression). This is why salt is used to deice roads in winter. However, even when cooled below the influence of solutes, water is only metastable and can remain liquid down to about –40 °C, at which point homogeneous nucleation occurs [15].

**Figure 2:**
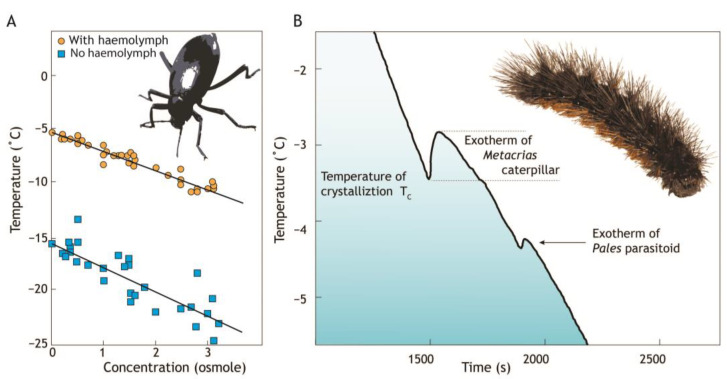
(**A**) The temperature of water crystallization is suppressed (freezing point depression) by increasing concentration of solutes such as sugars and salts (blue symbols). However, addition of haemolymph from *Eleodes blanchardi* beetles (orange symbols) [21,22] elevates this by about 10 °C. (**B**) The release of energy (exotherm) that occurs when water changes from the liquid to solid state (freezing) marks the temperature of crystallization (T_C_) or ‘supercooling point’. Detection of this exotherm in an experimental setting shows that ice has formed in the subject. In this example the caterpillar of the alpine tiger moth (*Metacrias huttoni*) has a higher (warmer) temperature of crystallization than its internal tachinid parasite, *Pales* sp. [23].

Heterogeneous nucleation caused by ice-nucleating agents is common in biological systems and can occur at temperatures just a few degrees below the freezing point depression (Figure 2A). When cooled, the aqueous solution in an insect body is in a metastable supercooled state where rapid ice growth occurs following nucleation. The degree of supercooling influences the properties of the ice crystals that form with low temperature nucleation, tending to result in small ice crystals that can penetrate tissues via gap junctions [16]. Thus, rapid freezing at low temperatures results in intracellular freezing that is more likely to cause damage [17,18]. By reducing the amount of supercooling, and thus the speed of subsequent crystal growth, heterogenous ice-nucleation in an insect can limit damage to insect tissues and render them freeze-tolerant.

The temperature at which the cooled bodily fluids freeze is the temperature of crystallization (T_C_) or nucleation temperature, and approximates to the supercooling point, although T_C_ is usually slightly lower. T_C_ can be measured because the phase change releases heat due to the exothermic nature of the process, and this can be detected as latent heat (Figure 2B). The temperature of crystallization of water inside terrestrial arthropods can be anywhere from −2 °C to −100 °C [19,20].

Elevation of the temperature of crystallisation can be detected in haemolymph extracted from insects (Figure 2), but the origin of the ice-nucleating (ice^+^) agents is unclear. Ice formation in freeze-tolerant insects is typically initiated by ice^+^ in the lumen of the gut and therefore outside of gut cells, and this facilitates slow ice growth after minimal supercooling [24]. Water is drawn out of cells, increasing the concentration of cryoprotective solutes by the freeze-concentration effect (Figure 2A), and lowering the freezing temperature within cells, thereby avoiding lethal intracellular freezing [19,25,26].

## 2. Ice-Nucleating Agents (Ice^+^)

The best agent of ice nucleation is another ice crystal, and some freeze-tolerant insects such as the North American woolly bear caterpillar (*Pyrrharctia isabella*), and linden bug (*Pyrrhocoris apterus*) undergo passive ice inoculation from outside their bodies [27,28]. In suitable circumstances this can result in survivable freezing above −3 °C. In many other cases biological particles and macromolecules act as very efficient ice nuclei (ice^+^), triggering ice formation at temperatures just below 0 °C. For example, snow makers on ski fields use ice^+^ particles harvested from bacteria (e.g., the rod-shaped, gram-negative bacterium *Pseudomonas syringae* in Snowmax^®^) to initiate crystallization of water [29].

Multicellular animals that can survive freezing, control ice formation in their bodies (usually extracellular water) using ice-nucleating (ice^+^) agents that arrange water molecules into stable embryo crystals at high sub-zero temperatures (−5 to −2 °C [30]) and limit supercooling [31]. At these relatively warm temperatures ice grows slowly, and the resulting large crystals limit stress to living tissue from solute freeze-concentration, and ice remains outside cells.

Ice recrystallization-inhibiting proteins are also found in some freeze-tolerant animals including some nematodes [32]. These proteins are thought to be involved in controlling the shape, formation, and stability of ice crystals after freezing (below 0 °C), by inhibiting recrystallization. At moderate sub-zero temperatures, a layer of liquid water sits between ice crystals, and larger crystals tend to grow at the expense of the smaller ones. Recrystallisation-inhibiting proteins limit this growth and thus limit damage to tissues.

## 3. Sources of Ice^+^ Particles

**Figure 3:**
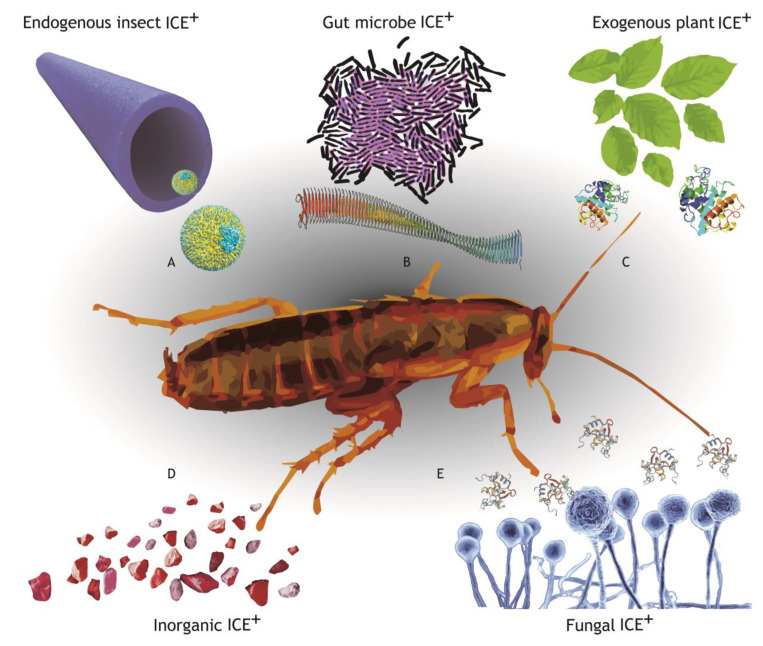
Insects that are freeze tolerant usually have relatively high temperatures of intercellular water crystallization facilitated by ice-nucleating agents (ICE^+^). There are many possible sources of ice+ agents in the environment and within the insect, of different sizes, stability, and efficiency. For example: (**A**) manufactured by the insect, e.g., lipoproteins in *Tipula trivittata* [46], (**B**) derived from microbes, e.g., large proteins from *Pseudomonas borealis* [47], (**C**) created by plants, e.g., class II chitinase [48], (**D**) environmental particles, e.g., kaolin-iron oxide composites [49], and (**E**) manufactured by fungi, e.g., fungal hydrophobins [50].

Ice-nucleating agents used by insects have many sources (Figure 3). Some freeze-tolerant arthropods produce specific endogenous protein ice-nucleators, and in other species selection has produced dual function protein ice-nucleators [19,33]. Ice**^+^** particles produced directly by insects are usually large, heat-sensitive proteins [19,34]. Insects freezing with a temperature of crystallization (T_c_) below −6 °C, as recorded by detection of an exotherm (See Figure 2), are likely to be producing their own (endogenous) ice^+^ [19,35]. Instead of manufacturing their own, some animals use exogenous ice^+^ particles for controlled freezing. Insects with a T_c_ between −6 and −3 °C might be using exogenous ice^+^ particles derived from the environment [36] (Figure 2B). However, more common sources of ice^+^ agents seem to be derived from bacteria and fungi (Figure 3) and some species of insect rely on commensal ice^+^ microbes for particles that facilitate high-temperature freezing [37,38].

It is increasingly apparent that ice^+^ microbes are environmentally common and widely influential, seeding ice crystal formation inside animals, on the surface of leaves [39] and in clouds [40]. Many cosmopolitan microbes have strains that produce ice^+^ proteins, and many of these ice^+^ lineages are plant pathogens that are abundant on leaf surfaces [39]. When temperatures drop at night, frost forms on the leaf stimulated by the ice^+^ proteins, and frost damaged leaves then become a resource for ice^+^ bacteria such as *Erwinia herbicola* and ice^+^ fungi such as *Fusarium* sp.

The bacterium *Pseudomonas syringae* produces large heat-sensitive ice^+^ particles on their cell membranes where the distance between particles dictates ice^+^ efficiency [41]. In contrast, other bacteria produce small ice^+^ particles and these still function as ice nucleators after heating (e.g., *Lysinibacillus* [40]). In some cases, sub-micron heat-sensitive ice^+^ particles (e.g., *Mortierella alpina* [42]) remain active long after the source dies (e.g., *Fusarium avenaceum* [43]) creating an environmental source of ice^+^ particles (Figure 3).

Many of the bacteria and fungi acting as ice^+^ agents are apparently found all over the world in many different habitats, but are they really the same? For example, the cosmopolitan fungus *Fusarium* is found in soils, and in water, on plants, and in clouds. When more than 100 strains of this fungus were studied to characterise their ability to catalyse ice formation, only ~16% of strains caused water crystallisation above −12 °C [44]. Strains that do not have the ability to act as ice-nuclei and raise the temperature of crystallisation are referred to as ice^−^ strains. The macromolecules (<100 kDa) produced by *Fusarium* are ice^+^ active and stable after the fungus has died, but they are heat sensitive and lose ice^+^ activity after reaching 40 °C. The bacterium *Pseudomonas syringae* is also ubiquitous [39] but includes both ice^+^ and ice^−^ strains [45].

## 4. Extended Genotype

The concept of the extended phenotype [51] considers the broadest influence of genes beyond their source organisms and it is recognised that the genetics of microbiomes considerably extends the evolutionary potential of their hosts [52]. The microbiome of an insect can complement its phenotypic scope in many ways. For example, insect microbiomes increase the range of food sources and growth rates of their hosts by improving the digestion of food, such as breaking down cellulose and increasing the absorption of nutrients. Recent studies are revealing this role in unexpected ways. In the digestive tract of the darkling beetle (*Zophobas morio*) larvae, live *Bacillus* bacteria that can decompose polystyrene, and free nutrients to the insect [53]. However, the influence of gut microbes on their host reaches beyond fundamental nutritional processes. Gut microbes are also involved in fat storage, production of essential amino acids, protection against pathogens with antimicrobial activity, and enhance the host’s immune response [54]. Some insects rely on cues from microbes for the initiation of metamorphosis from larvae to adult [55] and a gut bacterium is implicated in the manufacture of a pheromone that mediates cohesion of the gregarious phase of the locust *Schistocerca gregaria* [56]. The contribution of gut microbes to cold adaptation is now being recognized in a range of animal hosts [57]. For example, brown bears benefit from their gut microbes during hibernation, and mice inoculated with bear microbes gain a similar metabolic phenotype [58]. In insects, freezing is typically initiated in the gut [24,30,59], and ice^+^ activity is greater in the gut contents of many insects than in their haemolymph [60,61] (Figure 3). Thus, the gut and its microbiome may be the key to understanding freeze-tolerance in a range of invertebrates (Figure 3).

**Figure 4:**
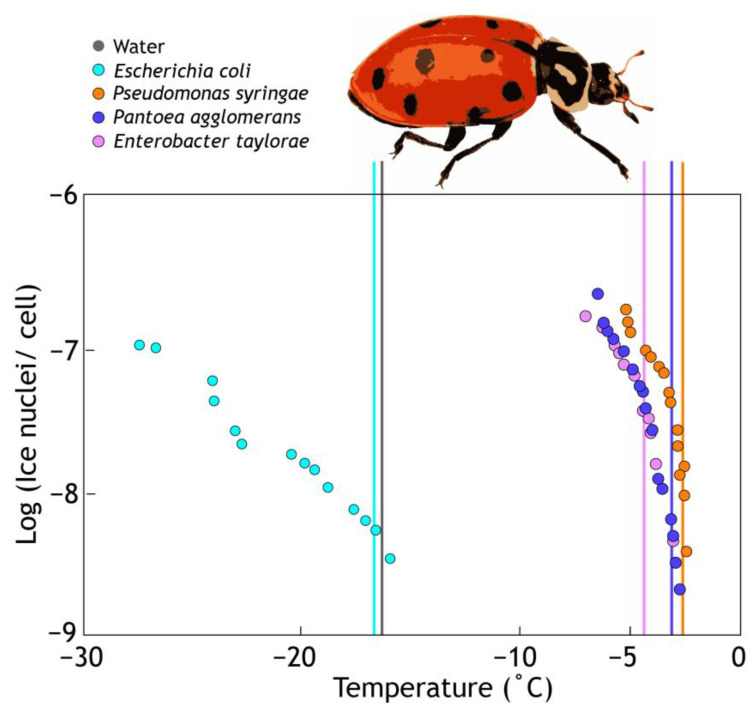
Ice-nucleating bacteria bring the temperature of crystallization of water droplets to close to 0 °C (spots) compared to pure water or water containing non-ice-nucleating bacteria (*E. coli*). When *Hippodamia convergens* beetles consume these same bacteria (*P. syringae*, *P. agglomerans* (formerly *Enterobacter agglomerans*), and *E. taylorae*), they freeze at a higher temperature (shown as vertical lines; see [30,35,65]).

Insects can shape their gut microbiota by ingesting food containing beneficial microbes or ice^+^ agents in food species [62]. For example, two freeze-tolerant beetles (*Hydromedion sparsutum* and *Perimylops antarcticus*) collected from the same sub-Antarctic island were both host to gut *Pseudomonas* sp. with ice^+^ activity [63]. Experimental work has demonstrated that insects fed ice-active bacteria froze at higher temperatures than pre-feeding. Normally *Hippodamia* ladybird beetles freeze at −16 °C but after experimental feeding with ice^+^ bacteria they froze at just −3.5 °C [35] (Figure 4). This results in their death as the beetles are not freeze-tolerant. This experimental system might be used for biological control to reduce the abundance of pest insects, but frost damage to crops at relatively high temperatures is a possible unwanted side effect of applying ice^+^ bacteria to plant leaves. In contrast, freeze-tolerant beetle and moth species play host to specific ice^+^ microbes that facilitate controlled high-temperature freezing allowing these insects to survive cold conditions [37,63,64].

## 5. Transmission of Insect Microbes

Microbes inhabiting insect guts are either inherited from parents or acquired from the environment. Many insect species have symbiotic relationships with bacteria in their gut, body cavity, or within cells of various tissues (Gupta and Nair 2020) [66], and to ensure that offspring are inoculated with these specialist microbial partners a range of mechanisms for vertical transmission (mother to offspring) of symbionts have evolved. Endocellular symbiotic bacteria that are essential for survival and reproduction of their host insect have stable maternal inheritance that has resulted in host–symbiont cospeciation (e.g., *Buchnera* of aphids and *Wigglesworthia* tsetse flies [67,68]). In several well-studied hemipteran systems (e.g., leafhoppers and aphids) specialist bacteria are distributed widely within the insect host. By colonizing organs such as the gut and male and female gonads they achieve vertical transmission and transmission between sexes during mating [66]. *Fusarium* fungus associated with sugarcane infects foraging caterpillars [69] and is subsequently transmitted in the eggs from mother to offspring. This pathogenic fungus manipulates both plant and insect to promote its infection and dissemination.

Bacteria that colonise only the insect gut can nevertheless be transmitted vertically in a number of ways [66] including egg smearing and coprophagy [70]. Plataspid stinkbugs use a symbiont capsule encasing their beneficial bacteria, this is deposited when the female lays her eggs. The newly hatched nymphs eat the capsule and their guts are colonized by their mother’s symbiotic bacteria [67,71]. In a similar manner, some grasshoppers obtain gut bacteria from their mothers who inoculate the foam covering their eggs with bacteria that their nymphs will ingest when hatching [72]. Cockroaches transmit obligate endosymbiont *Blattabacterium* bacteria via frass [73]. It is also possible for early instar insect nymphs to acquire symbionts directly from the surrounding environment, as seen in the bean bug *Riptortus pedestris*, which does not transmit its *Burkholderia* symbiont vertically from mother to offspring [68]. Thus, transmission of specific microbes in insects is feasible, and it is also possible that through the selection of foods containing desirable bacteria, mobile insects can shape their own gut microbiota and ice^+^ agents [62]. There is even potential for horizontal exchange of microbes between unrelated hosts and this could confer adaptive phenotypes resembling convergent evolution.

## 6. Freeze-Tolerant Insects in Aotearoa/New Zealand

**Figure 5:**
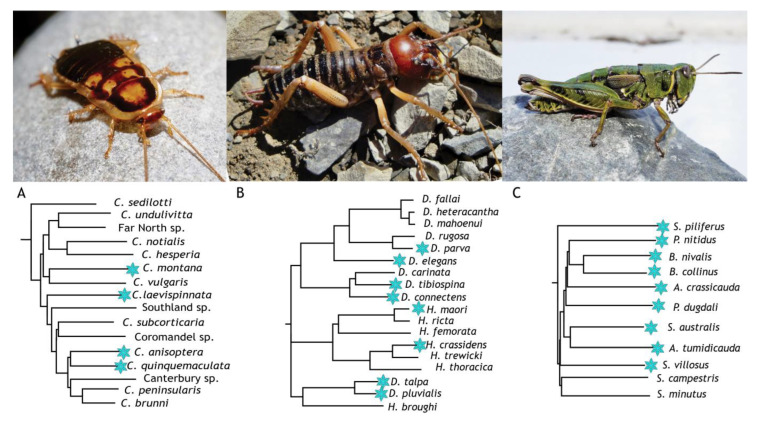
Three freeze-tolerant endemic Aotearoa-New Zealand insect species and their phylogenetic relatives. (**A**) The Otago alpine cockroach (*Celatoblatta quinquemaculata*) is a nocturnal omnivore. Alpine lineages are not monophyletic within New Zealand *Celatoblatta* (denoted with blue star on tree). (**B**) The mountain tree wētā (*Hemideina maori*) is a nocturnal omnivore. Alpine lineages are not monophyletic within the wētā clade. (**C**) The southern alpine grasshopper (*Sigaus australis*) is a diurnal herbivore within a cold-adapted endemic lineage. Phylogenetic relationships were reconstructed from (**A**) unpublished mtDNA genome sequences, (**B**) 755 transcriptomes [81], and (**C**) whole mtDNA genome sequences [82].

In the New Zealand alpine zone, a profusion of separate endemic insect lineages [74,75] can survive freezing, including cockroaches [76] (Figure 5a), wētā [77] (Figure 5b), grasshoppers [78] (Figure 5c), moths [23,79], and stick insects [80]. In these mountains temperatures can drop to freezing levels even in the summer, which is likely why these insects are freeze-tolerant throughout the year [10]. However, despite the frequent episodes of cold in all seasons, temperatures on New Zealand mountains rarely fall far below −10 °C. This may be important for the evolution of freeze-tolerance as the structure and growth of ice crystals continues to change as temperatures drop, putting greater stress on cellular organisms. Here we summarise some key features of three alpine insect species that are freeze-tolerant and are better understood in terms of their biology and freezing ability than other New Zealand alpine insects. For all three species that can be found at the same high-elevation sites in the Southern Alps details have been recorded of their internal temperature of ice crystal formation, the initiation site of freezing, their parasites, and their microbiome.

### 6.1. Celatoblatta quinquemaculata—The Otago Alpine Cockroach

This small alpine cockroach of Otago, New Zealand (maximum mass about 130 mg) (Figure 5A) lives among rocks and vegetation above ~800 m above sea level, where they are exposed to highly variable temperatures that even in summer can drop below freezing [83]. It is active all year and can survive freezing at any time of the year [76,83,84,85]. The genus *Celatoblatta* comprises a radiation of more than a dozen species endemic to New Zealand [86], and several of these live at a range of elevations. Some are restricted to cold environments; however, alpine adaptation does not appear to unite a monophyletic group of species (Figure 5).

**Figure 6:**
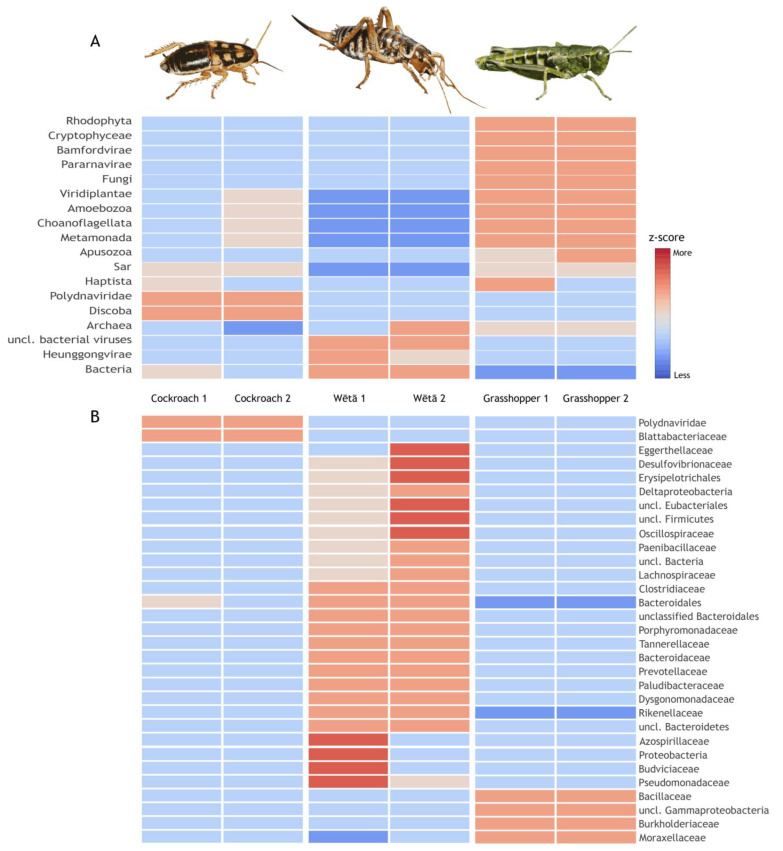
Gut microbiome of six New Zealand freeze-tolerant insects inferred from shotgun DNA sequencing. Heatmaps generated using MEGAN v6.24.1 [105] and visualised as a Z-score for the two samples from cockroaches (*Celatoblatta quinquemaculata*), wētā (*Hemideina maori*), and grasshoppers (*Sigaus australis*) from Central Otago. Data are clustered by sample composition similarity (above) and proportions of the data (right) showing all assigned nodes. The six samples were analysed using the Kaiju webserver (using the nr-euk 2021_03 database), downloaded and processed with a custom Perl script. Results are (**A**) visualised at the taxonomic level of kingdom with only data above a normalised cut-off of 1 read per million sequences shown, and (**B**) visualised at the taxonomic level of family with only data above a normalised cut-off of 100 reads per million sequences shown.

*Celatoblatta quinquemaculata* freeze at the relatively high temperature of −5.4 °C and have a lower lethal temperature of −8.9 °C [84] (Table 1). Although concentration of the cryoprotective agent trehalose fluctuates seasonally within these insects [59], the seasonal difference between the highest and lowest mean temperature of crystallization was only 1.8 °C [84]. The temperature at which *C. quinquemaculata* freeze (temperature of crystallization) is the same as the temperature at which some bacterial ice^+^ agents ensure ice formation occurs. Although ice^+^ agents have been found in insect haemolymph, *C. quinquemaculata* ice^+^ activity is strongest in the frass, gut contents, and gut [24,60]. Two-thirds of *C. quinquemaculata* specimens examined had the nematode *Blatticola barryi* in their gut [87], which is also freeze-tolerant. Microbial communities are known to be diverse in omnivorous cockroaches (Schauer et al., 2012) [88], and our preliminary DNA sequencing of gut contents from two *C. quinquemaculata* is no exception. In the DNA samples we detected high proportions of the expected obligate mutualistic endosymbionts from the family *Blattabacteriaceae* [89] (Figure 6).

Ice formation in this cockroach is thought to be intercellular, but gut cells of this species are unusual in their ability to survive intracellular freezing [18,59,90,91]. Because the gut is a closed system, the avenues available for ice to reach the haemolymph are limited, but a possible pathway is through the cells forming the wall of the gut. When freezing, 74% of an alpine cockroach’s body water is converted into ice [76]. Both thermal-hysteresis (the lagging of freezing) and ice recrystallisation-inhibition activities are absent from the haemolymph of *C. quinquemaculata*, although both types of chemical activity occur in its gut tissue [59]. Preliminary analysis of whole cockroach ice shell extracts showed evidence for three groups of ice-binding proteins; two small (8.4 kDa and 9.3 kDa) and one larger (>50 kDa; unpublished data).

### 6.2. Hemideina maori—The Mountain Tree Wētā

The southern hemisphere crickets known as wētā include the world’s largest freeze-tolerant insects—*Hemideina maori* and the alpine scree wētā *Deinacrida connectens* [61,77,92,93,94]. The phylogenetic relationships within the New Zealand tree and giant wētā reveal that cold adaptation is not restricted to a single clade (Figure 5B) and could plausibly be considered a shared ancestral state with some species having lost freeze-tolerance [61,93,95]. The mountain tree wētā has a wide distribution on the Southern Alps above the treeline (>1100 m asl) and some forest-free inland locations. This nocturnal alpine omnivore uses cavities beneath stones and crevices in rock outcrops to shelter during the day. It is freeze-tolerant throughout the year and ice-nucleation activity is found in the gut throughout the year (Figure 5B). It has a temperature of crystallization of −3.8 °C [24,77] and ice formation begins in the hindgut and may then propagate through the gut cells, eventually nucleating the haemolymph (Table 1). Some osmotic dehydration must occur ahead of the approaching ice front, but not enough to prevent freezing [24]. It was found that the maximum proportion of body water of an individual that can freeze (82%) was converted to ice when *H. maori* was held at −5 °C, with the freezing event taking approximately ten hours to complete [92]. Ice-nucleation activity is highest in the gut and frass of *H. maori* but is also evident in the haemolymph. The nucleation activity of the haemolymph is lost by heating or passage through a fine filter, suggesting that the nucleator is a large protein [85].

Some freeze-avoiding insects such as beetles and aphids have freeze-avoiding parasites that influence the temperature of crystallization of their hosts [96,97]. Individual *H. maori* wētā are typically parasitized by about 50 *Wetanema* sp. nematodes living in their hind gut [98]. *Wetanema* can survive freezing within its host but as it tolerates much lower temperatures than *H. maori* [98] it is unlikely that *Wetanema* provide the wētā host with ice^+^ particles. No significant difference was found between the temperature of crystallization of *H. maori* individuals with and without nematodes and there was no correlation between the number of nematodes and the temperature of crystallization of the host. The temperature of crystallization of the host and parasite differ in some insects such as caterpillars of the New Zealand eastern tiger moth, *Metacrias huttoni*, which reaches exotherm at −3.3 °C, well ahead of its internal tachinid fly parasite (*Pales tecta*) that freezes at −4.2 °C (Figure 2B) [23].

### 6.3. Sigaus australis—The Southern Alpine Grasshopper

The alpine grasshoppers of New Zealand can also survive freezing at any time of the year (Pers. Obs.) [78]. Although the twelve species of cold-adapted grasshoppers are monophyletic (Figure 5C), their common ancestor existed before the development of the New Zealand alpine environment (Figure 5) and so alpine adaptations in species of this clade appear to be the result of convergent evolution [82]. Convergent evolution could result from novel solutions to the same stress (e.g., as seen in New Zealand stick insects [80]). About a third of these grasshoppers have gregarine protozoans in their guts [99] which must also tolerate freeze thawing. In the lab, adult *Sigaus australis* have a temperature of crystallization recorded between −0.1 and −4.8 °C [100], suggesting the involvement of exogenous ice^+^ agents, with the medium temperature at which all available body water froze between −1 and −3 °C [78]. The lower lethal temperature for this grasshopper is −11 °C [5]. New Zealand *Chionochloa* tussock grasses produce ice^+^ proteins that initiate freezing between −5.2 and −6 °C [36] and potential plant ice^+^ protein genes have been identified for *C. macra* (e.g., *IRI2* and *EAF2*) [101] providing a valuable resource for the study of potential plant-derived ice^+^ agents. However, New Zealand alpine grasshoppers appear to eat little grass [102], and as the ice^+^ proteins of *Chionochloa* tussock grasses initiate freezing at a lower temperature than the grasshopper freezes, this is probably not a source of ice^+^ for *S. australis*.

## 7. Exploring the Microbiome

Each endemic New Zealand insect lineage may have independently evolved production of unique endogenous ice^+^ agents to control and tolerate freezing (Table 1), but freezing starts at temperatures above −6 °C in the gut, where high concentrations of ice+ particles occur. Is it plausible then, that a common environmental source of ice+ enables so many New Zealand insects to be freeze-tolerant?

Metagenomic DNA sequence data from the hindgut of the sympatric New Zealand alpine cockroach, wētā, and grasshopper have provided intriguing results. All three species are home to microbes with ice^+^ potential, including *Pseudomonas syringae*, *P. fluorescens*, *Mortierella* spp., and *Fusarium* spp. [103]. Any combination of these taxa could contribute to freeze-tolerance of the insect hosts. High-throughput sequencing of DNA extracted from the guts of two individuals of each insect species revealed complex microbial communities with significant differences in microbiome composition among host taxa but less variation among conspecific individuals (Figure 6a). Within the cockroach gut we detected abundant sequences from obligate mutualistic *Blattabacterium* that are known endosymbionts of this insect order. Within the wētā there was a high diversity of bacteria, including an abundance of Firmicutes as recorded in the congeneric *Hemideina thoracica* [104]. Despite the grasshopper and wētā being more closely related (both Orthoptera), the microbiome of the wētā and sympatric cockroach appear the more similar of the three host species. The grasshopper hindguts were relatively rich in viruses and eukaryotes (fungi, metamonads, and amoebozoans) compared to the wētā gut samples that were dominated by uncultured bacteria (Figure 6b).

If ice^+^ agents are of microbial origin as suggested by their high concentration in the gut and signal from preliminary DNA metagenomics (Figure 6), then there is the potential for different insect lineages to contain identical ice^+^ agents. One approach to exploring this would be to isolate ice-binding proteins using the ‘ice shell’ technique [106] and characterise them by mass spectrometry. Determination of amino acid sequences from digested protein fragments allows the linkage of proteins with their respective genes via databases such as Pfam/InterPro (https://www.ebi.ac.uk/interpro/ accessed on 3 November 2022). Motif matching can identify candidate ice-binding regions in novel sequences; however, with non-model organisms such as these insects there may be few homologues in existing databases and so matching against transcriptomes derived from each group of animals is likely to be important for protein characterisation.

RNA extracted and sequenced from gut contents can be used to assemble the meta-transcriptomes of host microbiomes. This allows the study of gene expression across a whole complex of microbial communities within their natural environment. Genes with specific roles can be identified [107], and the symbiotic role of the gut microbiota in insects explored [108]. As specific ice^+^ agents may constitute a relatively small proportion of expressed genes in each sample, enrichment for messenger RNA is an important step. For microbial studies subtractive, hybridization is generally used for depletion of ribosomal RNA, but use of biotinylated oligonucleotides specific to 5S, 16S, and 28S rRNA is an emerging option [109]. Insect transcriptomes can be subtracted from the microbial RNA and the open-reading frames from mRNA sequences interrogated for the distinctive motifs of candidate ice^+^ proteins. Both functional and compositional convergence of the microbiomes of different hosts is possible. Functional information can be extracted by profiling presence/absence and abundance of microbial pathways in a community, and gene ontology analysis will reduce complexity and highlight biological processes. The functional Kyoto Encyclopaedia of Genes and Genomes orthology (KEGG) database provides opportunities for studying functional convergence [110].

Meta-transcriptomics is used primarily for functional analysis of genes expressed in the gut microbiome but can also be used for comparing the taxonomic composition of the microbial community. Despite depletion, rRNA sequences are very abundant in all organisms and likely to be represented among data, and these suit taxonomic analyses of community composition [111,112].

Differential gene expression experiments comparing mRNA extracted from a range of tissue samples before and during freezing is a powerful tool for identifying ice^+^ agents. By mapping reads from each tissue sample before and after freezing to assembled transcriptomes [113], (after unique gene identification and normalizing read count) one can identify differential gene expression [80] and find out what microbes are actively transcribing at low temperatures. In this way it will be possible to identify the genes and organisms involved in freeze-tolerant phenotypes exhibited by a large array of New Zealand insects.

## Figures and Tables

**Table 1:** Comparison of critical temperatures for three alpine insects from Aotearoa-New Zealand.

Taxon	Species	Temperature of Crystallization °C	Lower Lethal Temperature °C	% of Body Water Frozen	Initiation of Freezing
Cockroach	*Celatoblatta quinquemaculata*	−5.4	−8.9	74	gut
Wētā	*Hemideina maori*	−3.8	−10	82	hindgut
Grasshopper	*Sigaus australis*	−4	−11	Not known	Not known

## Data Availability

The new data presented in this study are available on request from the corresponding author. The data are not publicly available as analyses have yet to be completed.
